# Parity and time reversal elucidate both decision-making in empirical models and attractor scaling in critical Boolean networks

**DOI:** 10.1126/sciadv.abf8124

**Published:** 2021-07-16

**Authors:** Jordan C. Rozum, Jorge Gómez Tejeda Zañudo, Xiao Gan, Dávid Deritei, Réka Albert

**Affiliations:** 1 Department of Physics, The Pennsylvania State University, University Park, PA 16802, USA.; 2 Eli and Edythe L. Broad Institute of MIT and Harvard, Cambridge, MA 02142, USA.; 3 Department of Medical Oncology, Dana-Farber Cancer Institute, Harvard Medical School, Boston, MA 02115, USA.; 4 Network Science Institute and Department of Physics, Northeastern University, Boston, MA 02115, USA.; 5 Channing Division of Network Medicine, Department of Medicine, Brigham and Women’s Hospital, Harvard Medical School, Boston, MA 02115, USA.; 6 Department of Molecular Biology, Semmelweis University, Budapest, Hungary.; 7 Department of Biology, The Pennsylvania State University, University Park, PA 16802, USA.

## Abstract

We present new applications of parity inversion and time reversal to the emergence of complex behavior from simple dynamical rules in stochastic discrete models. Our parity-based encoding of causal relationships and time-reversal construction efficiently reveal discrete analogs of stable and unstable manifolds. We demonstrate their predictive power by studying decision-making in systems biology and statistical physics models. These applications underpin a novel attractor identification algorithm implemented for Boolean networks under stochastic dynamics. Its speed enables resolving a long-standing open question of how attractor count in critical random Boolean networks scales with network size and whether the scaling matches biological observations. Via 80-fold improvement in probed network size (*N* = 16,384), we find the unexpectedly low scaling exponent of 0.12 ± 0.05, approximately one-tenth the analytical upper bound. We demonstrate a general principle: A system’s relationship to its time reversal and state-space inversion constrains its repertoire of emergent behaviors.

## Teaser

Fast analysis of attractor commitment in networks of stochastic switches answers a 50-year-old attractor scaling question.

## INTRODUCTION

Many complex systems in the natural, social, or technological realm exhibit emergent behavior, i.e., collective dynamics arising from the interaction of entities governed by simple rules (*1*–*4*). Examples include phase transitions (*5*, *6*), flocking (*7*), consensus formation (*8*), and spontaneous synchronization of oscillators (*9*). Modeling frameworks that are frequently used to study the collective behavior of individuals include nonlinear dynamics (*10*, *11*), agent-based models (*12*), cellular automata (*13*), and network models (*14*, *15*). Boolean models sit at the intersection of these approaches [e.g., (*16*–*18*)]. They assign a time-varying binary variable to each system entity, represented as a node in a network of interactions. They exhibit diverse long-term dynamics (attractors) that represent collective behavior (for example, consensus of individuals), and they describe the evolution toward an attractor (for example, consensus formation from an initially disordered state).

Boolean modeling of electronic circuits is well known (*19*), but many other familiar models can also be viewed as Boolean models. The quenched (zero-temperature) Glauber model, a dynamic variant of the Ising model, considers the dynamics of each atom’s two possible spin orientations under the influence of its neighbors (*20*, *21*). Another example category includes models of spreading binary opinions through social networks [reviewed in (*8*)]. The McCulloch and Pitts neural network model introduces a propositional logic of all-or-none neuronal activation (*22*); the Hopfield model also considers two activities for each neuron and assumes a complete network of interactions (*23*).

Boolean models are well suited to elucidate system-level decision-making, i.e., robust commitment toward one of the dynamical attractors in a multistable system. This has made their use especially widespread in biology. They were introduced by Kauffman (*24*) and Thomas (*25*) as prototypical models for gene regulatory networks that underlie cell fate decisions (such as those that happen during cell differentiation). A large body of research has since shown that the attractors of Boolean models correspond to cell fates or stable patterns of cell activity (such as the cell cycle). Boolean models integrate and encode current knowledge of a biological process, fill any gaps of knowledge with hypothesized interactions, and predict the behavior of the system under loss of function, constitutive activation, or external control of system entities (*25*–*27*). They are frequently used to study cell differentiation processes such as T cell specialization (*28*, *29*), developmental processes such as patterning during embryogenesis in *Drosophila melanogaster* (*30*, *31*), and cancer [e.g., metastatic reprogramming (*32*–*34*) and prediction of targeted therapies (*35*, *36*)]. Model predictions in a variety of systems were verified experimentally (*34*, *37*–*40*).

Alongside models of specific systems, analysis of the expected collective behaviors exhibited by generic Boolean models has also proven insightful. Ensembles of Boolean models [random Boolean networks (RBNs)] have been studied for decades [reviewed in (*41*–*43*)]. These ensembles exhibit an order-to-chaos transition as dynamical and topological parameters are tuned. In the intermediate (critical) regime, the ensembles exhibit features of biological cells, including stability against perturbations and plausible scaling laws for the number and size of attractors with the system size (see text S1 for details). Here, we answer a long-standing question about the scaling law in the presence of timing stochasticity (i.e., when the update order and timing of variables are stochastic).

Despite Boolean models’ discrete nature and apparent simplicity, it is nontrivial to connect dynamical properties of decision-making to the underlying interaction network. Brute-force exploration of their state spaces is not generally feasible. A typical Boolean model of a biological process with a few dozen variables has tens of billions of states. Genome-scale models can have thousands of variables, resulting in many more states (~10^300^ to ~10^9000^) than Planck volumes in the observable universe (~10^185^). This challenge has motivated decades of research analyzing discrete dynamics without exhaustive state-space searches (*44*), for example, by analyzing how feedback loops in the interaction network constrain dynamics (*45*, *46*). While the body of research regarding how network structure constrains dynamics has proven invaluable, note that multiple Boolean systems are compatible with each interaction network. Ambiguity can be eliminated by defining a network whose graph structure unambiguously represents the update functions that govern the time evolution of each variable. One such network representation, the expanded network (also called the logical or prime-implicant hypergraph) (*31*, *47*, *48*), defines two virtual nodes for each entity, denoting the two possible values of its binary variable. Connections among virtual nodes encode the update functions. The structure of this auxiliary network tightly constrains the attractors of a system (*33*, *49*, *50*). The expanded network can be used to identify control strategies that drive the system to a desired attractor (*51*, *52*). The most parsimonious of these control strategies involve determining the so-called driver node(s) of self-sustaining circuits (*53*, *54*). In this way, fixing the state of a few driver nodes ensures the system’s convergence into a target space from any initial condition. This type of analysis has been used, for example, to pinpoint key proteins in pathological cell processes (*51*, *53*, *55*). Although initially developed for Boolean models, the expanded network has been generalized to multilevel discrete and ordinary differential equation (ODE) analogs (*55*, *56*).

We go beyond the previous use of the expanded network and characterize each virtual node with a binary activity and impose a parity structure that facilitates the proof of new theorems about the effects of perturbations on system trajectories. We call this extended version of the expanded network the parity-expanded network. With these additions, the parity-expanded network is a complete representation of the Boolean dynamical system and is a dynamical system in its own right. In addition, we describe the time reversal of stochastically asynchronous Boolean systems and use it to identify subsets of the state space that cannot be reached from states outside the subset. Using parity and time-reversal transformations in tandem, we developed a new algorithm to efficiently identify all attractors of large-scale Boolean systems. We apply the algorithm to answer the long-standing question of how quickly the number of attractors in asynchronous RBNs increases with network size.

## MATERIALS AND METHODS

We begin by recalling relevant concepts from Boolean modeling and defining the notation we use throughout. Constructing a Boolean model usually starts with the synthesis of the modeled system’s interaction graph. An interaction graph is a signed directed graph whose nodes are the *N* entities of a system and whose edges represent positive (activating) or negative (inhibiting) influence. Each entity *i* is characterized by a variable *X_i_* that can take one of two values: 1 (“active”) or 0 (“inactive”). Each *X_i_* updates its value according to the output of an update function *f_i_* : {0,1}*^N^* → {0,1} that maps each system state ***X*** = (*X*_*i*_0__, …, *X*_*i*_*N*−1__) to either 1 or 0. Any Boolean function can be expressed algebraically using logical operators (e.g., “not,” “or,” and “and” with symbolic representations ¬, ∨, and ∧, respectively). There are several schemes for determining the timing of variable updates. We use the stochastic asynchronous update scheme, in which at each step, a single variable is randomly chosen to update its value (each variable must have a nonzero update probability; these are often chosen to be uniform). Compared with other updating schemes, this scheme removes spurious oscillations that arise from unrealistic perfect synchrony but otherwise preserves long-term behaviors (*57*–*59*). Once the update functions are determined and an update scheme is selected, the Boolean system is fully specified. Throughout this work, we use the stochastic asynchronous update scheme, and hence, the term “system” is to mean a set of *N* update functions *f_i_* together with the implicit stochastic asynchronous update scheme. Each Boolean system induces a state transition graph (STG) with 2*^N^* nodes that represent all possible system states and with directed edges from one node (system state) to another when the parent state can be updated in one time step to attain the child state. Under stochastic asynchronous update, each node has between 0 and *N* outgoing edges. The attractors of a Boolean system are the terminal strongly connected components of the STG (i.e., they have no edges that exit the component). They are divided into two types: point attractors (also called fixed points or steady states), which contain only one state, and complex attractors, which contain more than one state. The topology of the STG is not affected by biasing some nodes to update more frequently than others. Therefore, the attractor repertoire does not depend on the precise probabilities that individual nodes are selected for update in the stochastic asynchronous update, as long as the probabilities remain nonzero.

### A new framework: The expanded network through the lens of parity

The expanded network (also called the logical or prime-implicant hypergraph) (*31*, *47*, *48*) was introduced as an auxiliary network constructed from the Boolean update functions. The expanded network nodes represent Boolean literals (e.g., *X_i_* and ¬*X_i_*), and its hyperedges (generalized edges that connect sets of nodes) represent prime implicants [irreducible sets of regulator states that result in *f_i_*(***X***) = 1] of the update functions. Here, we introduce a new definition of the expanded network that uses parity-related concepts and highlights its role as an invariant of the parity transformation.

**Fig. 1:**
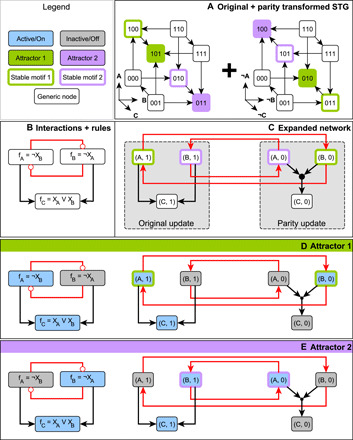
The relationship between the parity-expanded network and parity transformation illustrated on a three-variable Boolean system. The STGs are shown in (**A**); each system state is represented as the triple *X_A_X_B_X_C_*. Each state with fewer than three outgoing edges (state transitions) also has a self-loop, which is omitted for visual clarity. The interaction network and update functions are indicated in (**B**), and the parity-expanded network is indicated in (**C**). The system has two attractors, shown in (**D**) and (**E**), with blue nodes active and gray nodes inactive. States in the STGs of (A) are arranged so that the individual variables define coordinate axes. In this arrangement, the states form the corners of a cube. The parity transformation reflects each attractor through the center of this cube. The parity-expanded network in (C) has two parts or layers: regular virtual nodes (A,1), (B,1), and (C,1), whose activity updates according to the usual update functions, and negated virtual nodes (A,0), (B,0), and (C,0) that use the parity transformed update functions. The filled black circle represents the hyperedge from the set {(A,0), (B,0)} to (C,0) and indicates the AND operation in the update function of the virtual node (C,0). Positive regulation (black arrows) stays within a layer of the parity-expanded network, while negative regulation (red arrows) crosses between layers. Virtual nodes (A,1) and (B,0) form a stable motif (green outline), as do the virtual nodes (A,0) and (B,1) (purple outline). In any state of the system, half of the parity-expanded network is active. The stable motif (*A*,1), (*B*,0) describes the trap space containing attractor 1 and does not overlap with its parity transformed set (*A*,0), (*B*,1), which corresponds to the trap space containing attractor 2 (E).

The parity transformation acts on a Boolean system by the change of variables *X_i_* ↦ ¬ *X_i_* for all variables *X_i_*, mapping the original system with update functions *f_i_* to the system governed by update functions ¬*f_i_*. This mapping induces further transformations on any structure derived from the update functions, and so, in a slight abuse of notation, we say that the parity transformation acts on all these structures. For example, the parity transformation relabels the nodes of the STG so that all 1s become 0s and vice versa (see Fig. 1). Viewing these node labels as spatial coordinates (so states lie on the vertices of a unit hypercube), the parity transformation is the spatial inversion of this hypercube through its center (see Fig. 1).

Parity allows for a succinct definition and extension of the expanded network that builds upon the eponymous structure defined in (*48*). Here, we give an abbreviated discussion of this object and leave formal details to text S2. A parity-expanded network *G* is a dynamically endowed hypergraph. Each node *I* of the parity-expanded network, called a virtual node, is an ordered pair *I* = (*n*(*I*), *s*(*I*)) consisting of a system entity, in this context denoted *n*(*I*), and a value *s*(*I*), which is either the constant 1 or the constant 0. There are two virtual nodes associated with each system entity *i*, namely, (*i*,1) and (*i*,0); we call this pair of virtual nodes contradictory. A set of virtual nodes that does not contain any contradictory pair is called consistent. Each virtual node *I* is endowed with a Boolean variable σ*_I_*, whose time evolution is governed by an update function *F_I_*; this defines a 2*N*-dimensional dynamics that can be restricted to an *N*-dimensional subset such that *F*_(*i*,0)_(***X***) = *F*_(*i*,0)_((¬ ***X***, ***X***)) = ¬ *f_i_*(***X***) and *F*_(*i*,1)_(***X***) = *F*_(*i*,1)_(¬ ***X***, ***X***) = *f_i_*(***X***). In the context of this restriction, we may think of σ*_I_* as the indicator function for the subspace defined by *I*, i.e., we define σ_(*i*,0)_(***X***) = ¬ *X_i_* and σ_(*i*,1)_(***X***) = *X_i_*. We view the expanded network as having two layers: an “original update” layer and a “parity update” layer. This can be seen in Fig. 1, where the parity-expanded network nodes are partitioned into these two layers. In contrast with earlier versions of the expanded network and the logic hypergraph, the parity-expanded network is a dynamical system in its own right; its nodes are characterized with activity variables and a stochastic time evolution function ***F*** : {0,1}^2*N*^ → {0,1}^2*N*^, restricted to the *N* degrees of freedom of the underlying Boolean system. See text S2 for an example of ***F*** written explicitly as a function of 2*N* variables for the network of Fig. 1. A main advantage of this notation is that it allows us to treat the negated and non-negated versions of variables and functions simultaneously. In addition, the explicit endowment of the parity-expanded network with dynamical properties avoids awkward constructions along the lines of “the Boolean network corresponding to the parity-expanded network” in several places; rather, we can speak more simply of just “the parity-expanded network.”

The dynamics of these activity variables are encoded in the connectivity of the parity-expanded network. A hyperedge connects a set of parent virtual nodes *S* = {*I*_0_, *I*_1_, …, *I_k_*} to a target virtual node *J* if ∧_*I* ∈ *S*_σ*_I_* is a prime implicant of the update function for σ*_J_*, i.e., of *F_J_*. Pictorially, we represent hyperedges with more than one parent using intermediary “composite nodes,” which correspond to AND gates. For an example of a Boolean system and its parity-expanded network, see Fig. 1 (B and C). Hyperedges between and within parity layers encode important features of the dynamics. For example, negative influence manifests as interlayer hyperedges. Thus, it follows from theorem 19 of (*46*) that if a Boolean system’s interaction graph lacks negative feedback loops and has no paths of opposite sign between any two nodes, then there is a change of variables that disconnects the parity layers from one another.

Arguably the most dynamically important of the parity-expanded network’s topological structures are its stable motifs (*48*) and stable modules (*56*), which correspond to specific states of generalized positive feedback loops in the interaction graph (although we will define them on the parity-expanded network). These determine trap spaces in the dynamics, which are regions of the state-space characterized by a set of fixed variable values that, once attained by a trajectory, confine the trajectory to that region for all subsequent time steps. These generalize the notion of point attractors in that only a subset of the system’s variables is fixed in a trap space. Many of our formal results rely on recasting these structures in the parity view of the parity-expanded network as follows. A stable module *M* is a non-empty sourceless subhypergraph of the parity-expanded network such that *M* does not overlap (does not share virtual nodes) with its image under parity. A stable motif is a stable module that does not contain any smaller stable module; note that this implies that a stable motif is strongly connected. Because stable modules (and, thus, also stable motifs) are sourceless, every virtual node in a stable module *M* can be maintained in its active state by other virtual nodes in *M*, meaning that the activity of *M* is self-sustaining. Once *M* is activated, it cannot be inactivated except via direct override of its virtual node activities by direct external controls (as opposed to inactivation via the effects of upstream pathways). Thus, *M* describes a control-robust trap space in which the values of certain variables are stationary (*47*, *51*). The trap spaces corresponding to the activity of stable modules are exactly those considered by (*49*), with larger trap spaces (more states) corresponding to smaller stable modules (fewer constrained variables) and vice versa. See Fig. 1 for an example of the parity-expanded network and stable motifs.

We leverage the parity properties of the parity-expanded network to prove new results about driver node sets (*54*) and their relation to attractors. Formal statements and proofs are given in text S3. Recall that a set of virtual nodes is called consistent if it is disjoint from its image under parity [i.e., it contains no pair of nodes of the form (*i*,1) and (*i*,0)]. We say that a consistent set of virtual nodes *S* drives a virtual node *I* if *I* is consistent with *S* and if *F_I_*(*X*) = 1 for every attractor state ***X*** of the dynamics obtained by restriction to the states in which *S* is active. As a particular example, the vertex set of any stable motif drives itself (is self-sustaining). The set of all virtual nodes driven by *S* is called the domain of influence (DOI) of *S*, written as *DOI*(*S*). It follows from this definition that (with probability 1) trajectories with *S* initially active eventually either inactivate *S* or activate all of *DOI*(*S*). It is often useful to study the subset *DOI*(*S*) − *S* of *DOI*(*S*), which consists of all virtual nodes (*i*, *s*) ∉ S for which *X_i_* = *s* is fixed in all attractors of the dynamics restricted to the subspace defined by *S*. Similarly, DOI(*S*) in its entirety is the union of DOI(*S*) − *S* and the members of *S* driven by DOI(*S*) − *S*. We say that *S* is self-negating if there exists a subset *T* of DOI(*S*) − *S* such that DOI(*T*) intersects the image of *S* under parity (i.e., *T* drives a node that contradicts *S*). In such cases, *S* cannot be active in every state of any attractor. This definition of the DOI is closely related to concepts presented in (*54*).

Calculating DOI(*S*) can be difficult in general, so we focus instead on a commonly used and easily calculated subset of the driving relation: the logical domain of influence (LDOI). A set *S* of virtual nodes logically drives a virtual node *I* (which may or may not be in *S*) if there is a nontrivial multipath in the parity-expanded network from a subset of *S* to *I* with all virtual nodes in the path consistent with *S* (this requires that *I* is consistent with *S*). Here, a (nontrivial) multipath from a set *S* to a node *I* is a (non-empty) finite sequence of hyperedges {(*h*_parents,*i*_, *h*_children,*i*_) : *i* = 0,1, …. , *n*} such that (i) *h*_parents,0_ ⊆ *S* = *S*_0_, (ii) *h*_parents,*i*_ ⊆ S_*i*−1_ ⊆ *h*_children, *i*−1_, and (iii) *I* ∈ *h*_children,*n*_. Note that for *I* ∈ *S*, a consistent nontrivial multipath exists from *S* to *I* [i.e., *I* ∈ LDOI(*S*)] if and only if upon restriction to the subspace defined by *S* and percolation of constant values, *I* becomes active. The set of all *I* logically driven by *S* is called the LDOI of *S*, written as LDOI(*S*) [or LDOI(*i*, *s*) when *S* = {(*i*, *s*)} is of size one]. Intuitively, LDOI(*S*) corresponds to the variable values that become fixed after percolating *S* through the update functions and simplifying algebraically. As demonstrated in (*54*), if *S* is a subset of LDOI(*S*), then LDOI(*S*) contains the virtual nodes of a stable motif. If LDOI(*S*) contains a stable motif *M*, we say that *S* (logically) drives *M*.

Our main result in this section (theorem 1 in text S3) states that if an attractor contains a state for which *S* is active, it must also contain a state for which DOI(*S*) is active. This result illustrates how the DOI of a set of virtual nodes can constrain which states must coexist within an attractor. These considerations are important in constructing Boolean models in which certain system configurations should correspond to different long-term qualitative system behaviors (e.g., phenotypes). Two corollaries of this result allow one to study the conditions under which an attractor avoids activating a particular set of stable motifs. This problem is of interest, for example, in biology, where one or more stable motifs may correspond to a diseased state of the system; the goal in this case is to identify drug targets that avoid stabilizing the diseased state. We will also make use of these results in later sections to enumerate a system’s attractors.

The first of the two corollaries (corollary 1 in text S3) can be viewed as a compatibility condition for a stable motif’s activity and the activity of a virtual node that drives it. In the context of system control, it states that if fixing *X_i_* is sufficient to eventually activate a stable motif *M*, then the oscillation of *X_i_* is also sufficient to activate *M*. It can also be viewed as a consistency condition for attractors: If *X_i_* = *s* leads to activation of *M*, then we cannot have *X_i_* = *s* in an attractor (even transiently) in which *M* is inactive. This result provides a powerful way to identify circumstances in which no stable motif activates: In such cases, all stable motif drivers must be permanently inactive. Specifically, we collect all single-node drivers of all stable motifs into a set Δ and test whether or not ¬Δ = (*i*, ¬ *s*) : (*i*, *s*) ∈ Δ is self-negating. If it is self-negating, then it follows that at least one element of ¬Δ is not permanently active in each attractor, and thus (by corollary 1), this element must eventually stabilize at least one stable motif. The formal statement of this result is given as corollary 2 in text S3.

### Time reversal of Boolean systems and stable motifs of the time-reversed system as unstable “Gardens of Eden”

**Fig. 2:**
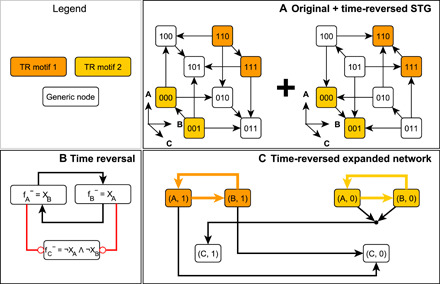
Illustration of the time-reversal transformation on the example from Fig. 1. (**A**) The original system’s STG alongside the STG of its time reversal. The time reversal has the effect of reversing the direction of each edge in the STG. The interaction network and update functions of the time-reversed system are depicted in (**B**) alongside its parity-expanded network in (**C**). Red interaction network edges indicate inhibition, while black edges indicate activation. The virtual nodes and hyperedges of the time-reversed system’s two stable motifs are highlighted in yellow and orange in the parity-expanded network, and the regions of state space in which they are active are highlighted in the system’s STG on the left in (A). Note that the system states highlighted in yellow (000 and 001) viewed in the original system’s STG form a subgraph that has no in-component and the state 001 is a Garden of Eden state. The same is true for the system states highlighted in orange. In this example, the sign of the regulation (inhibition versus activation) is reversed under the time reversal. This holds, in general, for all interactions except self-regulation, which does not change sign under time reversal.

A second set of foundational results in this work is the construction of the time reversal of an asynchronous-update Boolean system, which exists despite the system’s inherent stochasticity. We use the time-reversal transformation to help identify discrete analogs of unstable manifolds in the state space. The time reversal (TR) of a Boolean system governed by update functions *f_i_* is called the time-reversed system and is governed by the update functions *f_i_*^−^, where *f_i_*^−^ = ¬ *f_i_*(*X_i_* = ¬ *X_i_*) (i.e., in a state ***X***, the value of *f_i_* is obtained by negating the value of *X_i_*, evaluating *f_i_*, and taking the negation of that output). Like the parity transformation, we formally define the time reversal as a transformation of the Boolean system given by its effect on the update functions. It similarly induces transformations of structures that are derived from these functions. For example, the time reversal of a parity-expanded network *G* for update functions *f_i_* can be obtained as the parity-expanded network for the update functions $f_{i}^{-}$ and is denoted TR(*G*). Similarly, the STG of a system is related to its time-reversed counterpart by reversing the direction of all edges (see Fig. 2A). Thus, one may follow the evolution of the time-reversed system on the STG by following edges in the reverse direction. Equivalently and in analogy to concepts in solid-state physics, one may imagine that all but one of the nodes in the STG are occupied by walkers who may not share an STG node. If the walkers randomly follow the edges of the STG, then the position of the unoccupied “hole” evolves according to the possible trajectories of the time-reversed system.

The concept of Garden of Eden states, which are source nodes of the STG (*60*, *61*), can be generalized as subgraphs of the STG that do not have incoming edges; we call these subgraphs Garden of Eden spaces. They are analogous to unstable manifolds of ODE systems in the sense that no trajectory can enter these spaces from the outside. Any trap space of a system, and, in particular, any of its stable motifs, is a Garden of Eden space in the time-reversed system and vice versa. For example, the states marked in green in Fig. 1A form a trap space of the original system and a Garden of Eden space of its time-reversed counterpart, while the states marked in yellow form a trap space of the time-reversed system and a Garden of Eden space of the original system. An important consequence of this time reversal–based mapping between trap spaces and Garden of Eden spaces is that no attractor of a system can cross the boundary of any of the system’s trap spaces or Garden of Eden spaces. This observation is especially helpful in eliminating states from consideration when searching for attractors via direct STG construction or in reducing the number of relevant initial conditions for study. We leverage these methods to construct an efficient attractor-identification algorithm and explore their utility by way of example in the “Application to decision-making in empirical biological network models” section.

### Stable motif succession determines state-space decision-making and attractors

**Fig. 3:**
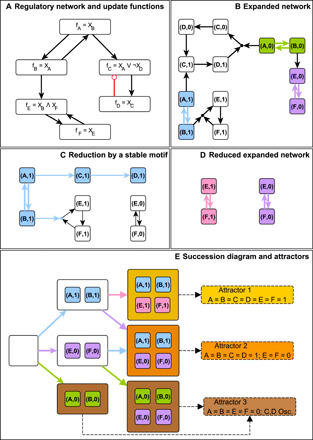
Outline of the iterative stable motif reduction process on a simple example. (**A**) A Boolean system’s interaction network and update functions. The corresponding parity-expanded network, *G*, is shown in (**B**); there are three stable motifs, highlighted in blue, green, or purple. In (**C**), the stable motif corresponding to *X_A_* = *X_B_* = 1 is selected and the effect of maintaining its activity is highlighted in blue; in particular, it leads to *X_C_* = *X_D_* = 1. The variables that are unfixed (*X_E_* and *X_F_*) then form the bistable system Red(*G*, {(*A*,1), (*B*,1)}) whose parity-expanded network is shown in (**D**). The reduced system has two stable motifs, highlighted in pink or purple; the latter was also a stable motif of the original system. All possible sequences of stable motif selection and reduction are summarized in a succession diagram (**E**). Each node of the succession diagram is a set of virtual nodes that contains the stable motifs that were selected for use in the reduction. The colors of the edges in the succession diagram indicate which stable motif is selected to get from one reduction to the next. The process terminates when no stable motifs remain, and the attractors of the maximally reduced systems are identified as attractors of the unreduced system (highlighted in yellow, orange, and brown).

As a Boolean system’s STG has 2*^N^* nodes and up to 2*^N^N* edges (for stochastic asynchronous update), for systems with many entities (large *N*), it is impractical to use the STG to determine the system’s attractor repertoire. Stable motif–based, or trap space–based, attractor identification methods are often more effective (*47*, *48*). In the iterative approach of (*48*), a system’s stable motifs are identified, and one is selected to “lock in.” The system’s update functions are then reduced under the assumption that the system’s state is confined to the region described by the stable motif, resulting in a reduced network. Rephrased in our framework, each stable motif is selected, in turn, and the parity-expanded network is simplified under the assumption that the motif’s virtual nodes are active, resulting in a reduced Boolean system. We use the notation Red(*G*, *M*) for the reduced parity-expanded network that results after restricting the dynamics to the subspace defined by the activity of *M* and its LDOI. The process is repeated recursively for each reduced parity-expanded network until all possible permutations of stable motif activation are explored. The result is a succession diagram, Σ, which is a directed acyclic graph whose nodes are the unions of the vertex sets of stable motifs used to obtain each reduced system (see text S2 for a formal definition). For an example of this process and the resulting succession diagram, see Fig. 3.

The succession diagram serves as a summary of the decisions in the system dynamics that lead to successively more restrictive nested trap spaces. Each node of the succession diagram corresponds to a region of the state space in which the denoted stable motifs are active. Each branch point in the succession diagram represents potential choices to be made; which choice is ultimately selected by the system depends on various factors including the stochastic update order. It follows from the nestedness of these trap spaces that the system cannot transition between regions that are not connected by a path in the succession diagram, i.e., the succession diagram encompasses the entire repertoire of decisions that the system is capable of making. The close relationship between the succession diagram and branch points in the dynamics is illustrated in fig. S1 by constructing the full STG of the example from Fig. 3. In the “Application to decision-making in empirical biological network models” section below, we illustrate how the succession diagram can be used to analyze the complex state-space decision-making in systems biology models.

**Fig. 4:**
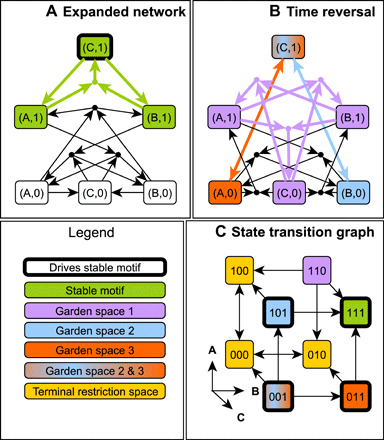
An illustration of methods to refine the portion of the STG that may contain motif-avoidant attractors using the system *f_A_(*X*) = f_B_(*X*) = ¬X_A_∧¬X_B_∨X_C_; f_C_(*X*) = X_A_∧X_B_* as an example. (**A**) The system’s parity-expanded network and its sole stable motif (in green). This stable motif has one driver set, {(C,1)} (bold outline). By corollaries 1 and 2 (text S3), any motif-avoidant attractor must have *X_C_* = 0 and *F*_(*C*,0)_ = ¬ *X_A_* ∨ ¬ *X_B_* = 1 fixed, i.e., such an attractor can only contain states that satisfy *R*(***X***) = ¬ *X_C_* ∧ (¬*X_A_* ∨ ¬*X_B_*) = 1. (**B**) The parity-expanded network of the time-reversed system, with its three stable motifs highlighted in purple, blue, and orange. (**C**) The STG of the system. The states corresponding to each of the stable motifs in (A) and (B) are highlighted in matching colors. States with (C,1) active are highlighted with a bold outline; none of these states can be part of a stable motif–avoidant attractor. The three stable motifs of the time reversal partition the STG into five subgraphs based on which time-reversal stable motifs are active: none, purple, blue only, orange only, or both orange and blue. These subgraphs, highlighted by color, are Garden of Eden spaces of the forward-time system. No attractor of the forward-time system can cross between these regions. Any motif-avoidant attractor of the system must reside in the terminal restriction space of the STG (yellow). The states 100, 000, and 010 form a motif-avoidant attractor in which *X_C_* = 0 is fixed while *X_A_* and *X_B_* oscillate.

One must take special care to consider the possibility of oscillations that avoid activation of stable motifs. For example, consider the system shown in Fig. 4

$$f_{A}(X)=f_{B}(X)=\neg X_{A}\wedge \neg X_{B}\vee X_{C};f_{C}(X)=X_{A}\wedge X_{B}$$

This system’s parity-expanded network contains only one stable motif, the hypergraph induced by (*A*,1), (*B*,1), (*C*,1), which corresponds to the system’s sole point attractor *X_A_* = *X_B_* = *X_C_* = 1. Previous stable motif– or trap space–based methods (*48*, *49*) would correctly identify this point attractor by finding the corresponding stable motif. This system, however, contains an additional attractor in stochastic asynchronous update. In the second attractor, *X_C_* remains in the 0 state, while *X_A_* and *X_B_* oscillate; this second attractor is not identified by previous iterative stable motif reduction methods. Other existing methods can identify this attractor via simulation or can detect that at least one attractor lies outside the union of identified trap spaces. For a detailed discussion of the nature of these oscillations and their implications for the completeness of the attractor repertoire identified by iterative stable motif reduction, see (*51*). These oscillations motivate us to propose more robust automated methods that can identify oscillations that fail to activate stable motifs. These methods make practical what previously was impractical: the identification of the attractor repertoire of ensembles of large Boolean systems. Our method automatically identifies all complex attractors, including those that were overlooked by previous iterative stable motif reduction methods.

### Overview of the attractor identification algorithm

We follow an iterative stable motif approach to attractor identification in which stable motifs are recursively used to produce reduced Boolean systems (and corresponding reduced parity-expanded networks) until no additional stable motifs remain (see Fig. 3). At each stage in the iteration for which a reduced parity-expanded network contains a stable motif, we identify complex attractors that do not activate any additional stable motifs. We call such attractors motif-avoidant and call reduced systems with motif-avoidant attractors terminal. Although terminality requires analysis of the system’s STG in the general case, it is usually possible to substantially reduce the computational burden by application of a necessary condition for terminality that arises from corollary 2 (text S3) and properties of the parity-expanded network. We consider the set Δ of all virtual nodes that individually drive any stable motif to obtain theorem 3 (text S3), which can be informally stated as follows: All motif-avoidant attractor states satisfy *R*(***X***) = 1, where *R*(***X***) = ∧_*I* ∈ ¬ Δ_(σ*_I_*(***X***) ∧ *F_I_*(***X***) ∧ (∧_*J* ∈ LDOI(*I*)_σ*_J_*(***X***))) [and *R*(***X***) ≡ 1 when Δ is empty]. This result is a necessary consistency condition for the inactivity of stable motif drivers. In the example in Fig. 4, there is only one single-node driver of the system’s sole stable motif, namely, (*C*,1). Thus, the entire negated driver set is ¬Δ = (*C*,0). We calculate that LDOI(*C*,0) is empty and that the update function for (*C*,0) is *F*_(*C*,0)_(***X***) = ¬ *X_A_* ∨ ¬ *X_B_*. Therefore, we find *R*(***X***) = ¬ *X_C_* ∧ ( ¬ *X_A_* ∨ ¬ *X_B_*). In Fig. 4C, only the three yellow states have *R*(***X***) = 1, and so, any motif-avoidant attractor is confined to those three states.

We also leverage time reversal in determining terminality. If a stable motif *M*^−^ of the time-reversal TR(*G*) of the parity-expanded network *G* is active in a state *X*, then *X* can only be in an attractor of *G* if *M*^−^ (viewed as a set of virtual nodes in the forward-time system) is not self-negating in *G*. Thus, when considering states that may be in a motif-avoidant attractor of *G*, we can ignore states belonging to stable motifs of *G* and belonging to self-negating stable motifs of TR(*G*) and states for which *R*(***X***) is zero. We call the remaining states the terminal restriction space of *G*. For example, the terminal restriction space of the system in Fig. 4 is the yellow portion of its STG. In practice, focusing on the terminal restriction space can reduce the number of variables that must be simulated by a considerable amount. For example, we analyzed the 60-node T-LGL (T-cell large granular lymphocyte) network of (*62*), whose STG is too large (hundreds of petabytes or more) to practically construct. Previous stable motif–based algorithms can estimate the attractor repertoire in a matter of hours but cannot guarantee the absence of motif-avoidant attractors without building the STG (*48*). Exploring only the terminal restriction space, however, results in a reduction of the search space by a factor of over 2 billion and allows us to exactly identify all the attractors of the system in a matter of minutes [for the purposes of making explicit comparisons to a fully constructed STG, we analyze a simplified model (*63*) of this same system, in text S6]. We have also found many examples of RBNs (*K* = 2, *p* = 0.5, *N* = 4096) in which the terminal restriction space of the initial network is of size zero, a state-space reduction of 2^4096^. For a description of the algorithm that we used to efficiently search the reduced terminal restriction state space for motif-avoidant attractors, see text S5.

**Fig. 5:**
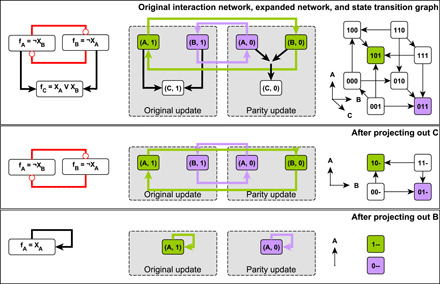
Deletion projection example. Each panel shows the interaction network, parity-expanded network, and STG of a system before projection (**top**), after projecting out one variable (**middle**), and after projecting out two variables (**bottom**). The attractors of each system and the stable motifs that are active therein are highlighted in green and purple. Attractors and stable motifs project onto attractors of the same color. The projection procedure preserves point attractors exactly. In general, the number of complex attractors (in this case, zero) serves as an upper bound on the number of complex attractors in the unprojected system [see, e.g., (*64*)]. One may view the action of the projection on the STG as contracting the top four nodes (100, 101, 110, and 111) to a single node (1--), with representative state 101 (transitions among these four nodes ultimately lead to 101); a similar view can be taken of the bottom four nodes. We note that the projection procedure respects the parity layer partitioning of virtual nodes. In accordance with theorem 2, the stable motifs [{(A,1),(B,0)} and {(A,0),(B,1)}, which project to the self-activating virtual nodes (A,1) and (A,0), respectively] are preserved in the sense that, for example, the attractor with (A,1) active in the bottom panel corresponds to an attractor in which the stable motif {(A,1),(B,0)} [which projects onto (A,1)] is active. This implies that the value of *X_A_* is sufficient to determine which attractor the system attains.

In some cases, even the terminal restriction space is too large to simulate. In these cases, we can obtain information about the number of motif-avoidant attractors via the network reduction method of (*64*, *65*). Variables that do not self-regulate are iteratively “deleted” by substituting their update functions into their successors’ update functions (see Fig. 5). This method, which we call deletion projection, provides a projection map, π, that has been proved to preserve certain features of the attractor repertoire, regardless of the number of deletions performed to obtain the projection map. In particular, all point attractors are preserved, and complex attractors of the original system map to one or more complex attractors of the projected system. Although not necessary for any of our theoretical results, variables with constant update functions are always prioritized for deletion as a computational optimization. It was shown in (*65*) that the order in which variables are deleted does not affect π. The inverse map preserves all point attractors but only a subset of complex attractors in general (in other words, the projected system can have more, but never fewer, complex attractors than are present in the unprojected system). We combine the concepts of stable motifs and driver nodes with the deletion projection method of (*64*, *65*) to investigate the terminality of a motif-reduced Boolean system. We show that the DOI of a set of virtual nodes that specifies a state for every variable in the projected system has a DOI in the unprojected system that specifies exactly one state, called a “representative state” in (*64*) (lemma 1 in text S3). Combining this result with theorem 1 (text S3) leads to one of our main results of this section: The activity of stable motifs in attractors is preserved by the deletion projection (theorem 2 in text S3). Theorem 2 allows us to test the terminality of a system by testing the terminality of its projection.

We give a visual overview of the algorithm in fig. S2. We also present a “by hand” example of the approach on a five-variable system in fig. S3. We implement the techniques described in this section in the StableMotifs Python Library (available at https://github.com/jcrozum/StableMotifs/). Notably, our attractor-finding method outperforms the earlier stable motif–based method of (*48*) and the boolSim tool (*66*) integrated into GINsim (see text S8) (*67*). For example, our code was able to compute the succession diagram of the 69-node unstimulated epithelial-to-mesenchymal transition model of (*33*) in under 2 min on a consumer-grade desktop, while the software implementation of (*48*) was unable to do so within 12 hours.

## RESULTS

### Application to decision-making in empirical biological network models

In this section, we illustrate how our methods can be applied to validated Boolean models of biological networks to analytically connect regions of state space to decision-making (points of no return) in their dynamics and to subnetworks (stable motifs) in the underlying interaction network. The biological network models that we focus on are a model of the mammalian cell cycle phase switch (presented in the subsection below) (*68*) and a model (*63*) of a type of white blood cell cancer (T-LGL leukemia) (presented in text S6). In particular, we identify and characterize the Garden of Eden spaces, illustrate an informative partitioning of the state space, and fully describe the attractor basins. The phase switch is a tristable molecular circuit that drives mitosis. Its three steady states mark three stages of the cell cycle: G_1_, G_2_, and the spindle assembly checkpoint (SAC). Under various biologically relevant conditions, such as coupling to other molecular circuits, the phase switch oscillates between these stages in the order that they occur in the cell cycle (*50*); here, however, we study this switch in isolation. Further details of this model, including the update functions and stable motifs, are given in text S7.

**Fig. 6:**
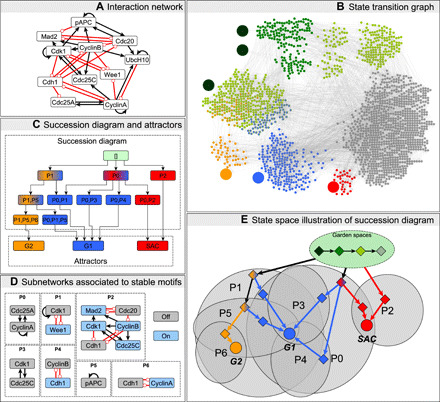
Parity and time-reversal analysis elucidates state-space decision-making in the phase switch model of (*68*). Text S7 provides further details about the model, including its update functions. The interaction network is presented in (**A**). The full STG is indicated in (**B**). The three point attractors of this model are the three large nodes highlighted in blue, yellow, and red and correspond to the G_1_, G_2_, and SAC phases of the cell cycle. The basins of attraction exclusive to each attractor are highlighted in the matching color. Gray nodes have paths to multiple attractors; the path taken depends on the stochastic update order. The stable motifs of the time-reversed system identify unstable Garden of Eden regions of the state space (highlighted in green) that cannot be (re)entered from the outside. A darker shade of green indicates the overlap of multiple Garden of Eden spaces. The three large green nodes are the Garden of Eden states of the system and are obtained analytically as the fixed points of the time-reversed system. (**C**) The stable motif succession diagram of the system, with stable motifs of the network and reduced network denoted by labels P0 to P6. The colors indicate, by the same scheme as in (B), which attractors are possible after commitment to each stable motif. (**D**) The subnetworks and node states associated with these stable motifs (blue, active/on; gray, inactive/off). These subnetworks and their states are obtained as strongly connected components of the parity-expanded network that do not contain parity-invariant subgraphs. In (**E**), we illustrate how the succession diagram and garden spaces together describe (analytically) the possible decisions that the system can make during its dynamics. Ovals represent specific subsets of the state space.

Our focus is on how details of a system’s decision-making capacity can be explored by analyzing the system’s time reversal in conjunction with the succession diagram. We emphasize insights gained from the stable motifs of the time-reversed system. In principle, more granular initial condition tracing is possible via the full succession diagram of the time-reversed system. Figure 6 shows how the succession diagram and Garden of Eden spaces provide a concise summary of the irreversible commitments in state space; this is especially useful when the STG is too large or too dense to analyze directly (compare Fig. 6B and Fig. 6E). The compressed state space illustration of Fig. 6E unites information from the original and time-reversed system. It is equally or more informative than the full STG (Fig. 6B). The green oval stands for Garden of Eden spaces, with the internal diamonds representing generic states in several categories of these spaces: The darkest green diamond represents Garden of Eden states (fixed points of the time-reversed system); the green diamond represents states in the overlap of multiple stable motifs in the time-reversed system; the light green diamond represents states in a single stable motif of the time-reversed system; and last, the gray diamond represents states that do not lie in any stable motif of either the forward or backward time system. That states of this last type belong in a Garden of Eden space follows from the definition of stable motifs and the lack of motif-avoidant attractors in this system. Progression from darker to lighter green represents the commitment to exiting Garden of Eden spaces. Gray ovals represent overlapping spaces determined by the stable motif written inside each oval. The diamond symbols in these overlaps mark relevant trap spaces, e.g., the yellow diamond marks the trap space in which motifs P1, P5, and P6 are all active. Each edge corresponds to an irreversible commitment to a smaller trap space. For example, the yellow-blue diamond marking the intersection of the P1 and P5 spaces has two edges: The edge to the yellow diamond indicates a transition to the intersection of the P1, P5, and P6 motif regions, which determines an irreversible commitment to G_2_ (entry into the exclusive basin of attraction to G_2_), and the edge to the blue diamond indicates a transition to the intersection of the P0, P1, and P5 motif regions, which determines an irreversible commitment to G_1_ (entry into the exclusive basin of attraction to G_1_). Circular symbols indicate the attractors and highlight their position in the narrowest trap space. The graph structure in Fig. 6E is isomorphic to the graph structure in Fig. 6C, demonstrating that the stable motif succession diagram encapsulates the trajectory of the system in state space. The compressed succession diagram representation has the benefit that it can be constructed for very large networks whose STG cannot be built. As we will demonstrate in the next section, we can build succession diagrams for networks whose STGs have more nodes (≈2^4000^) than can physically exist in the observable universe (≈2^600^, assuming one state per Planck volume and disregarding graph edges).

An important feature of this analysis is that decisions can be ascribed to specific strongly connected subgraphs of the interaction network. These subgraphs and their stable states correspond exactly to stable motifs, i.e., their representations in the parity-expanded network do not have any parity-invariant subgraphs. These can provide important biological insights. For example, because there are no paths in the succession diagram from P1 to SAC (Fig. 6C), we see that the P1 stable motif cannot be active during the SAC phase. Driver node analysis of the cyclin-dependent kinase 1 (Cdk1)–Wee1 feedback associated to the P1 stable motif (Fig. 6D) indicates that knockout of Cdk1 alone blocks the SAC. Following P1 commitment, the choice between G_1_ and G_2_ is decided by the activity of the Cdc25A-CyclinA feedback loop (P0) in competition with the Cdh1 (cadherin 1)-CyclinA feedback loop (P6) when pAPC (phosphorylated adenomatous polyposis coli complex) is inactive (P5).

### RBN results

As an additional application, we study the scaling of the average number of attractors of ensembles of RBNs generated by the Kauffman *N* − *K* model (*24*). In this model, each of *N* nodes receives *K* edges from randomly selected “regulator” nodes. Each node’s (quenched) update function randomly maps each of the 2*^K^* possible combined regulator states to 1 with probability *p* or to 0 with probability 1 − *p*. Tuning the indegree *K* or the activation bias *p* yields an order-to-chaos transition at 2*Kp*(1 − *p*) = 1 (in the thermodynamic limit, when *N* → ∞) (*41*, *69*–*71*). For additional details about previous studies of this model, see text S1. We address and resolve the open problem of the attractor number scaling in the critical *K* = 2, *p* = 0.5 regime under stochastic asynchronous update. The number of attractors provably scales as a power law asymptotically bounded above by *N*^ln4^ (*72*). In this section, we obtain the current best numerical estimate of the scaling exponent value (see Supplementary Code and the Random Boolean Network Application folder at https://github.com/jcrozum/StableMotifs/ for details on ensemble generation and analysis).

We generate ensembles for increasing network size *N* (from size *N* = 2 to size *N* = 4096) and apply our attractor identification algorithm to construct the succession diagram for each network and to determine or bound (in networks that are unusually computationally difficult) its number of attractors. For greater than 96% of the more than 10,000 unique networks generated, we are able to exactly enumerate the attractors, and this fraction never falls below 86% (260/300 for *N* = 2048) for any value of *N* considered here. For all but 13 (0.1% of the total) of the networks without an exact attractor count, we can constrain the number of attractors by using the deletion projection and/or counting the number of maximal stable modules (see Supplementary Code for details and implementation). Of these 13 networks, the plurality (*5*) is of size 4096, corresponding to 1.67%of the 300 networks generated for this size. For each of these remaining 13, we use the trivial lower bound of 1 for the number of attractors and choose an upper bound 10% larger than that of the most attractor-rich networks of the same size (see Supplementary Code for details). This makes these networks outliers without introducing undue sensitivity.

**Fig. 7:**
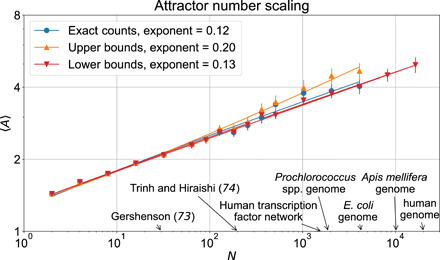
Summary of RBN attractor scaling fits. Symbols indicate the measured number of attractors and the lines represent fits of the form 〈*A*〉 = *a* + *bN^c^* using nonlinear least-square fitting (see Supplementary Code for implementation details). The fits yield intercept *a* = − 0.38, *a* = 0.44, and *a* = − 0.16 for the exact counts, upper bounds, and lower bounds, respectively. The exponents of the fits (*c* in 〈*A*〉 = *a* + *bN^c^*) are reported in the legend; see the main text or Supplementary Code for bootstrapped confidence intervals. For reference, the sizes of previously considered asynchronous RBN ensembles (*73*, *74*) and several genetic networks from biology (*79*–*83*) are annotated on the horizontal axis.

The scaling of the average number of attractors 〈*A*〉 as a function of network size *N* is shown in Fig. 7. By fitting power law 〈*A*〉 = *a* + *bN^c^*, we find that the exponent is *c* = 0.12 ± 0.05 [1 SD; 95% confidence interval (CI) [0.04,0.22]] when fitting only the networks for which we are able to exactly enumerate the attractors (blue circles and curve on Fig. 7). The attractor upper bounds (orange symbols and curve) yield an upper bound on the scaling exponent of *c* = 0.20 ± 0.05 (95% CI [0.11,0.30]). To ensure that the considered networks are sufficiently large, we analyze the scaling of the number of maximal stable modules in networks as large as *N* = 16,384 (recall that stable modules are source-free subgraphs of the parity-expanded network that have no parity-invariant subgraphs). Because maximal stable modules correspond to disjoint trap spaces in the state space, their number serves as a lower bound on the number of attractors. We include these lower bounds in our analysis of the lower bound scaling. In practice, the lower bounds are very often in 1-1 correspondence with the number of attractors, as is supported by the good agreement between the exact count of attractors and the lower bounds on the attractor counts. In particular, the scaling of the lower bounds (*c* = 0.13 ± 0.04, 95% CI [0.06,0.21]) is consistent with the attractor scaling for *N* ≤ 4096 and continues at least until *N* = 16,384, providing strong support that we have probed sufficiently large networks. These networks are of comparable size to many frequently studied genomes (e.g., *N* ≈ 4000 for the *Escherichia coli* genome, while for the human genome, *N* ≈ 20,000). Furthermore, our analysis of these lower bounds increases our confidence that we have considered sufficiently large *N* for the exact count scaling. This is because the mean estimates obtained by fitting up to *N* = 4096, *N* = 8192, and *N* = 16,834 are all in agreement with one another (*c* = 0.13 ± 0.04 for each of them) and within less than 10% of the mean estimate obtained for the *N* = 4096 exact counts (*c* = 0.12 ± 0.05). All the scaling exponents are well below the initially conjectured square root scaling (*c* = 0.5) and the theoretical maximum of *c* = ln 4(≈1.39). For additional details regarding the fitting procedure and error estimation, see Supplementary Code.

Overall, we obtain the best current estimate of the exponent of *K* = 2, *p* = 0.5 Kauffman networks under stochastic update. Our analysis represents an 80-fold increase in network size over previous exact or near-exact enumeration analysis of asynchronous *N* − *K* RBNs (*73*, *74*). We find a significantly lower exponent than the original exponent found by Kauffman and those identified in other stochastic updating schemes (*72*–*74*).

## DISCUSSION

Two central questions in complexity science are “what emergent behaviors could a complex system exhibit?” and “what controls a complex system’s selection of one emergent behavior or another?”. While interesting questions from a purely theoretical perspective, they also have important applications in engineering, social science, and medicine. The past several decades have seen a growing number of collaborations between biologists, computer scientists, mathematicians, and physicists that approach these questions using Boolean networks. This work continues that interdisciplinary tradition using geometric intuitions from physics to prove new results in the mathematics of Boolean dynamics, which we have applied to develop improved computational methods for analyzing complex systems common in biology and other sciences. Coming full circle, these methods have yielded new results in the statistical mechanics of RBNs. Our methods allow efficient identification of the subspaces of the state space where robust commitments happen and connect these decision-making spaces to subnetworks in the underlying interaction network. Time reversal identifies a previously unexplored type of decision-making: the commitment to exit a Garden of Eden space, eliminating the option to ever return to that space. We associate each decision with the stabilization or destabilization of strongly connected subnetworks that do not contain any parity-invariant parts.

The parity symmetry of Boolean systems has led us to propose the parity-expanded network. Much like the STG, it is parity invariant (up to node relabeling) and completely encodes the system’s dynamics, but its number of nodes grows linearly with the system dimension rather than exponentially. It unites large knockout and knock-in (constitutive activity) perturbations within the same framework, greatly simplifying their analysis compared to the similar ideas presented in (*31*), upon which we build. This earlier construction made use of three types of nodes and lacked explicit dynamical structure, requiring careful case splitting and multiple system representations to study system perturbations [see e.g., (*54*)]. Rather than distinguish nodes that correspond to on and off states at a definitional level, we distinguish these nodes by their behavior under a global parity transformation. This view elucidates the role of the parity-expanded network as a parity invariant and of its stable motifs as strongly connected components that have no parity-invariant subgraphs. These insights have enabled our formal proofs of novel results that relate network reduction methods, driver sets, and stable motifs, results that we have leveraged to develop a fast attractor-finding method.

We have presented the time reversal of a stochastic update Boolean system and demonstrated its usefulness in analyzing the forward-in-time dynamics. Our implementation is closely related to GINsim’s model reversal (*67*). Just as stable motifs of a system describe stable spaces (subspaces that trajectories cannot exit) in the dynamics, stable motifs in the time reversal of that system describe unstable spaces (subspaces that trajectories cannot enter). This observation is especially helpful in eliminating states when searching for attractors via direct STG construction or in reducing the number of relevant initial conditions for study. In addition, it demonstrates an important property: The activity of any stable motif of a system or its time reversal is constant for any attractor. In this way, time reversal and parity elucidate the “attractor-conserved” quantities of a system’s dynamics. These attractor-conserved quantities are only conserved within attractors and may initially vary. Nonetheless, it is notable that there is a well-defined notion of time reversal in these inherently stochastic systems and that it yields asymptotic conservation laws.

By combining new results stemming from the parity-expanded network and time-reversal construction, we developed a method for fast attractor identification in stochastic update Boolean systems. We used this new method to explore the scaling in attractor number for asynchronous *N* − *K* Kauffman networks. Using these methods, we probed the power law scaling of the *K* = 2 critical (*p* = 0.5) Boolean networks under asynchronous update. Our new techniques allowed us to find or bound the number of attractors in these networks for sizes larger than ever before considered (*N* = 16,384), and we cover much of the biologically relevant ranges of gene regulatory network sizes. The power law scaling that we observe is much lower (by a factor of about 10) than the theoretical maximum of ln4 (*72*) and also lower than the originally conjectured 0.5 scaling exponent (*24*). The low average number of attractors that we find (〈*A*〉 ≈ 4 for networks with *N* ≈ 4000) is consistent with previous results on the average number of point attractors and stable synchronous attractors in this ensemble (*57*, *61*). Notably, the low attractor number and slow scaling appear even when upper bounds for attractor numbers are used in the calculations instead of exact attractor counts, meaning that the results are not explainable by a systematic undercounting of attractors in large networks. Extending the considered network size beyond *N* = 4096 for the more easily computed lower bounds on the attractor size does not change the scaling exponent estimate. Because the exact counts and the lower bounds are in close agreement, this increases our confidence that we have considered sufficiently large *N* for the exact counts of attractors.

The relatively slow growth of the average number of attractors compared to (i) the originally conjectured 0.5 scaling exponent and (ii) the current cell type scaling estimate of 0.70 [0.88/1.26 = 0.70, based on the experimental data from (*43*)] has several possible explanations that suggest follow-up investigations. One possibility is that timing-specific attractors contribute substantially to the cell type scaling, implying that gene-regulatory synchrony plays a crucial role in a cell’s ability to differentiate. Alternatively, the scaling of the attractor number under stochastic update might vary between critical RBN ensembles (e.g., it may differ in ensembles using canalizing functions or threshold functions), and some of these other ensembles could more accurately reproduce the observed cell type scaling. Analysis of the attractor number scaling in other RBN ensembles using our approach should help answer this question. It is also possible that gene regulatory networks of living organisms have evolved to increase the number of robust attractors, a process that is not fully captured by these ensembles of RBN.

The methods developed here can be readily applied to the numerous published Boolean models of biological systems to elucidate their full attractor repertoire. Our framework can also bring further insight into a variety of models that could be reformulated as Boolean models. For example, the quenched Glauber dynamics set on a network (*75*), models of binary opinion propagation (*8*), or the Hopfield model (*23*) can be expressed with threshold Boolean functions. The stable motifs of these systems, and correspondingly the trap spaces of their dynamics, can be identified as particular instances of strongly connected subgraphs. In the Watts model of opinion propagation, the percolation of an opinion depends on the existence of a strongly connected subgraph of early adopters, who can be influenced by a single neighbor to adopt the opinion (*18*). We expect that future adaptation of our methods to these models will be able to reveal rare attractors (metastable states).

Apart from the direct application to Boolean models that we have emphasized here, time reversal and parity play a role in describing the fundamental logical relationships between entities in complex systems more generally. In this view, the logical parity inversion and time reversal of a system describe a coarse-grained and discretized version of the dynamics, which, in turn, provides insight into the dynamics of more detailed models; see e.g., (*45*, *76*) for further discussion. While the extent to which our key results generalize beyond the stochastic Boolean systems presented here remains an open question, we are encouraged by preexisting analogs of expanded networks in multilevel systems (*55*) and ODEs (*56*), as well as by results connecting logic-based models to ODEs (*45*, *76*–*78*). Although our focus here is at the level of interaction logic, our results suggest a new approach to analyzing complexity: studying the relationship of a complex system to its logical parity inversion and time reversal to constrain the system’s repertoire of emergent behaviors.

## SUPPLEMENTARY MATERIALS

Supplementary material for this article is available at http://advances.sciencemag.org/cgi/content/full/7/29/eabf8124/DC1

## Appendix

View/request a protocol for this paper from *Bio-protocol*.
